# Editorial: The impact of alkalizing the acidic tumor microenvironment to improve efficacy of cancer treatment, volume II

**DOI:** 10.3389/fonc.2024.1542787

**Published:** 2025-01-14

**Authors:** Reo Hamaguchi, Noha Mousaad Elemam, Shinji Uemoto, Hiromi Wada

**Affiliations:** ^1^ Clinical Cancer Research Team, Japanese Society on Inflammation and Metabolism in Cancer, Kyoto, Japan; ^2^ Department of Clinical Sciences, College of Medicine, University of Sharjah, Sharjah, United Arab Emirates; ^3^ Shiga University of Medical Science, Otsu, Japan

**Keywords:** cancer metabolism, tumor microenvironment, alkalization therapy, cancer treatment, Warburg effect, pH

The acidic tumor microenvironment (TME) is a hallmark of solid tumors and plays a critical role in cancer progression, metastasis, and resistance to therapy (1). This metabolic adaptation allows cancer cells to survive in oxygen-deficient but nutrient-rich environments, a phenomenon first described by Otto Warburg in 1956, who noted their reliance on glycolysis rather than oxidative phosphorylation for energy production (2). Subsequent studies, such as those by Gatenby and Gillies (3), have elaborated on how these adaptations are driven by Darwinian selection pressures, while Seyfried (4) further emphasized that cancer can be understood as a metabolic disease arising from disrupted cellular coordination. This condition arises from the high metabolic activity of cancer cells, which rely on aerobic glycolysis (the Warburg effect) to produce energy, leading to the accumulation of acidic metabolites such as lactate, contributing to an extracellular acidic environment while maintaining an alkaline intracellular pH (5). Enzymes such as carbonic anhydrase IX (CAIX) and proton transporters like the sodium/proton (Na^+^/H^+^) exchanger isoform 1 play key roles in stabilizing this pH imbalance, fostering tumor survival and growth (6–8). The extracellular pH in cancer cells, typically between 6.2 and 6.8 compared to the extracellular pH of 7.2–7.4 in normal cells, promotes invasive behaviors, enhances metastatic potential, increases resistance to therapy, and impairs the function of T cells and natural killer cells while promoting the activity of immunosuppressive cell types, such as regulatory T cells and myeloid-derived suppressor cells (9–12). Despite the critical role of TME acidification in cancer biology, most conventional therapies fail to target this unique metabolic feature, underscoring the need for new treatment strategies that address the pH imbalance and metabolic dependencies of cancer cells to improve therapeutic outcomes.

The clinical application of strategies targeting the acidic TME is of paramount importance. Alkalization strategies, such as pH-responsive nanomedicine, hold promise for improving drug delivery and efficacy by exploiting the acidic TME (13). Proton pump inhibitors, which increase extracellular pH, have shown potential in overcoming chemotherapy resistance (14). Additionally, targeting pH-sensing G protein-coupled receptors (GPCRs) may provide novel pathways to modulate tumor growth and immune interactions (15). Alkalization therapy, as buffer therapy using alkalizing agents, has also been explored in clinical settings, further highlighting the potential of pH modulation as a therapeutic intervention against the acidic TME (16, 17). Building on this evidence, a recent phase I/II trial found that combining an alkalizing agent with chemotherapy, particularly with S-1 as third- or fourth-line therapy, may improve survival in metastatic pancreatic cancer patients, reinforcing the potential of alkalization strategies in cancer treatment (18).

Following the success of “The Impact of Alkalizing the Acidic Tumor Microenvironment to Improve Efficacy of Cancer Treatment – Volume I”, this Research Topic continues to explore the therapeutic implications of alkalizing the acidic TME, focusing on uncovering mechanisms, developing innovative therapeutic strategies, and assessing their clinical applications. For instance, one study featured in Volume II has advanced our understanding of how the acidic TME interacts with tumor-specific gene expression and influences therapeutic outcomes. Kato and Mawatari demonstrated the heterogeneity of prognostic impacts associated with acidic conditions in melanoma, emphasizing the need to consider tumor type-specific responses in future research (Kato and Mawatari). Similarly, Gastelum et al. highlighted how intracellular acidification in multiple myeloma cells can overcome resistance to hypoxia-induced apoptosis, revealing a potential avenue for therapeutic intervention (Gastelum et al.). Research by Li et al. examined the heterogeneity of the tumor microenvironment in lung adenocarcinoma and demonstrated how these differences influence the choice of surgical approaches, highlighting the value of personalized treatment strategies (Li et al.). Meanwhile, Bogdanov et al. provided compelling evidence for the efficacy of alkalization therapy using sodium bicarbonate in a murine model of malignant ascites, showing prolonged survival and reinforcing the therapeutic potential of targeting the acidic TME (Bogdanov et al.). Guo and Wang further expanded on the role of the tumor microenvironment by systematically analyzing the immunosuppressive landscape of pancreatic ductal adenocarcinoma (PDAC). Their work revealed the intricate immunosuppressive networks within the TME, identifying key factors related to PDAC progression and resistance to therapy. This study lays the groundwork for innovative immunotherapy strategies aimed at overcoming these challenges (Guo and Wang).

This Research Topic highlights the critical role of the acidic TME in cancer biology and its impact on treatment resistance, emphasizing the potential of alkalization therapies to improve therapeutic outcomes. While significant progress has been made, challenges remain, including the heterogeneity of the tumor microenvironment and patient-specific responses. Further research is essential to address these complexities and translate these findings into clinical applications. By integrating molecular insights with innovative therapeutic strategies, we can move closer to more effective and personalized cancer treatments.
